# Supplementary material for: A kingdom in decline: Holocene range contraction of the lion (Panthera leo) modelled with global environmental stratification
Source: PeerJ. 2021 Feb 15;9:e10504. doi: 10.7717/peerj.10504 (PMC7891088; doi:10.7717/peerj.10504)
Supplement: Supplemental Information 1 [file peerj-09-10504-s001.docx]

**A kingdom in decline: Holocene range contraction of the lion (Panthera leo) modelled with Global Environmental Stratification.**

*David Cooper, Andrew Dugmore, Andrew Kitchener, Marc Metzger, Antonio Trabucco*

**Appendices**

*Appendix S1: High resolution figures of Global Environmental Zones for the present day, mid-Holocene, and Last Glacial Maximum ……………………………...***pages 3-6**

*Appendix S2 Reducing multiple global climate models to a single map output*

………………...............................................................……………………...**pages 7-10**

*Appendix S3: MaxEnt Model Parameters and Results…………………...* **pages 11-13**

*Appendix S4: All Lion Suitability Models for the Present Day, Mid-Holocene and Last Glacial Maximum………………………………………………………………***pages 14-16**

*Appendix S5 Saharan Rock Art reference table*……………………………**pages 17-18**

*Appendix S6: Modis Landcover Classes Table and GEnS/MODIS Comparison Figure*……………………………………………………………………………**pages 19-22**

**Appendix S1:**

**High resolution figures of Global Environmental Zones for the present day, mid-Holocene, and Last Glacial Maximum**

The datasets presented here are available at http://hdl.handle.net/10283/3274


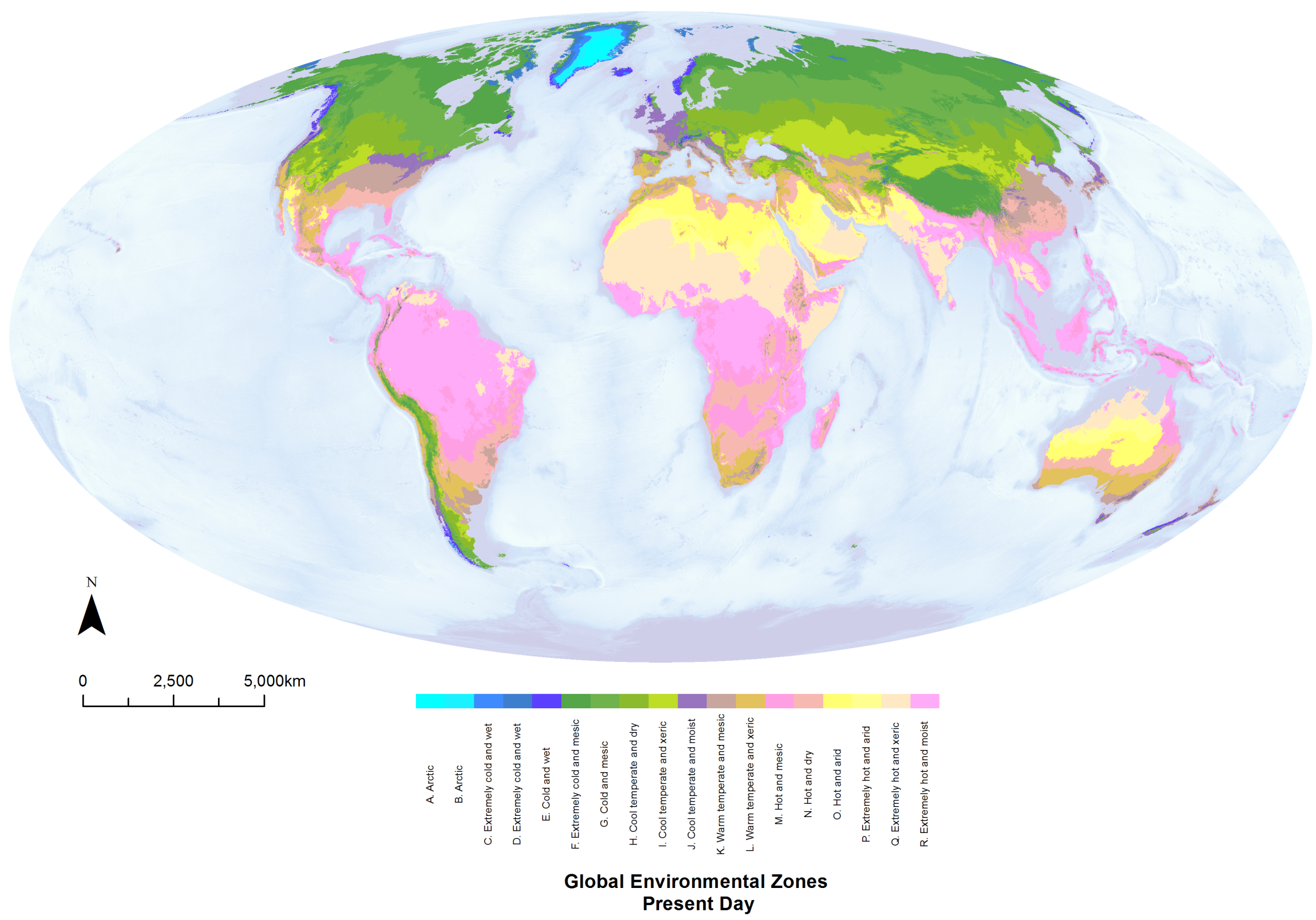

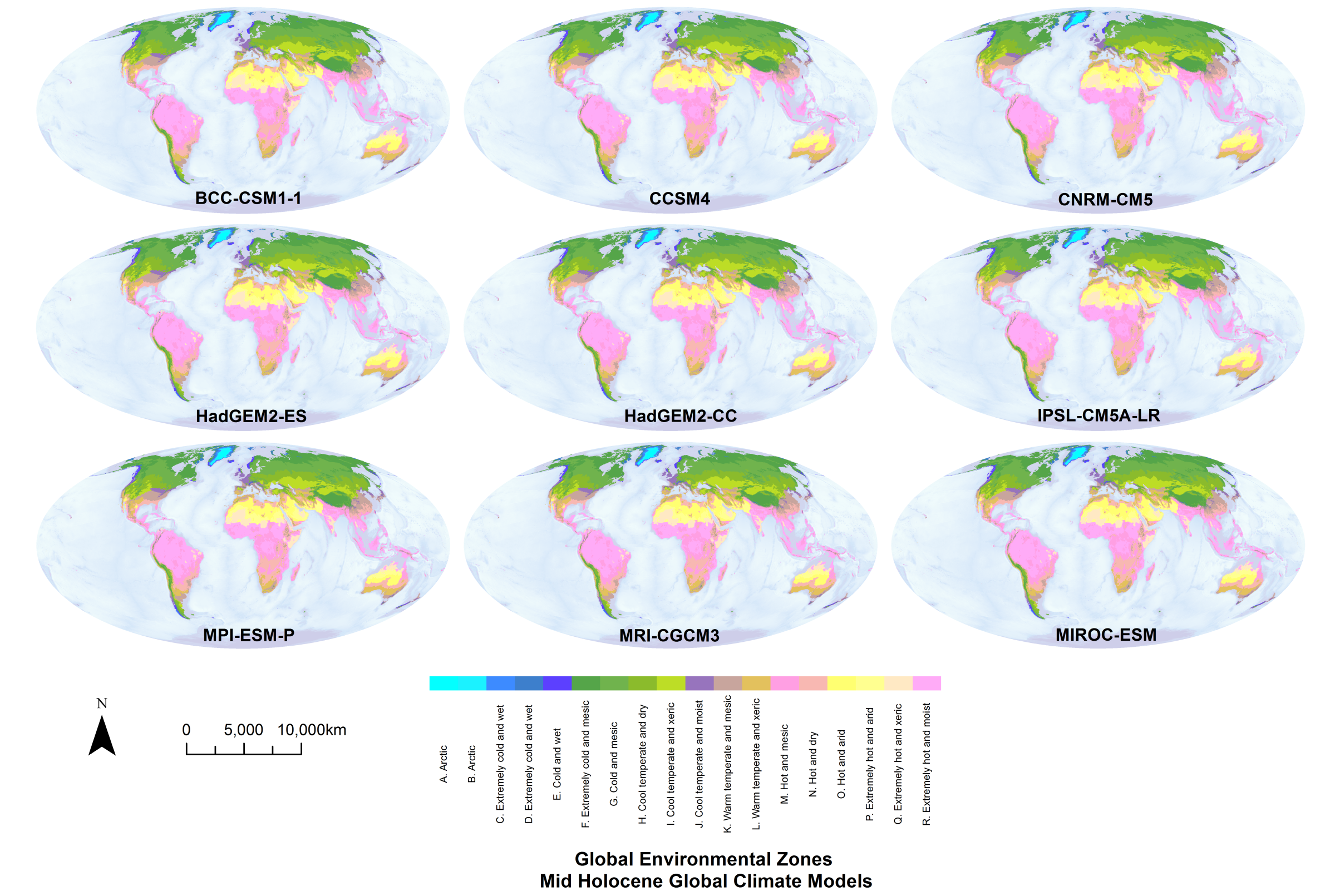


**Figure S1.1:** Global Environmental Zones for the present day.

**Figure S1.2:** Global Environmental Zones for the mid-Holocene, based upon nine coupled general circulation models.


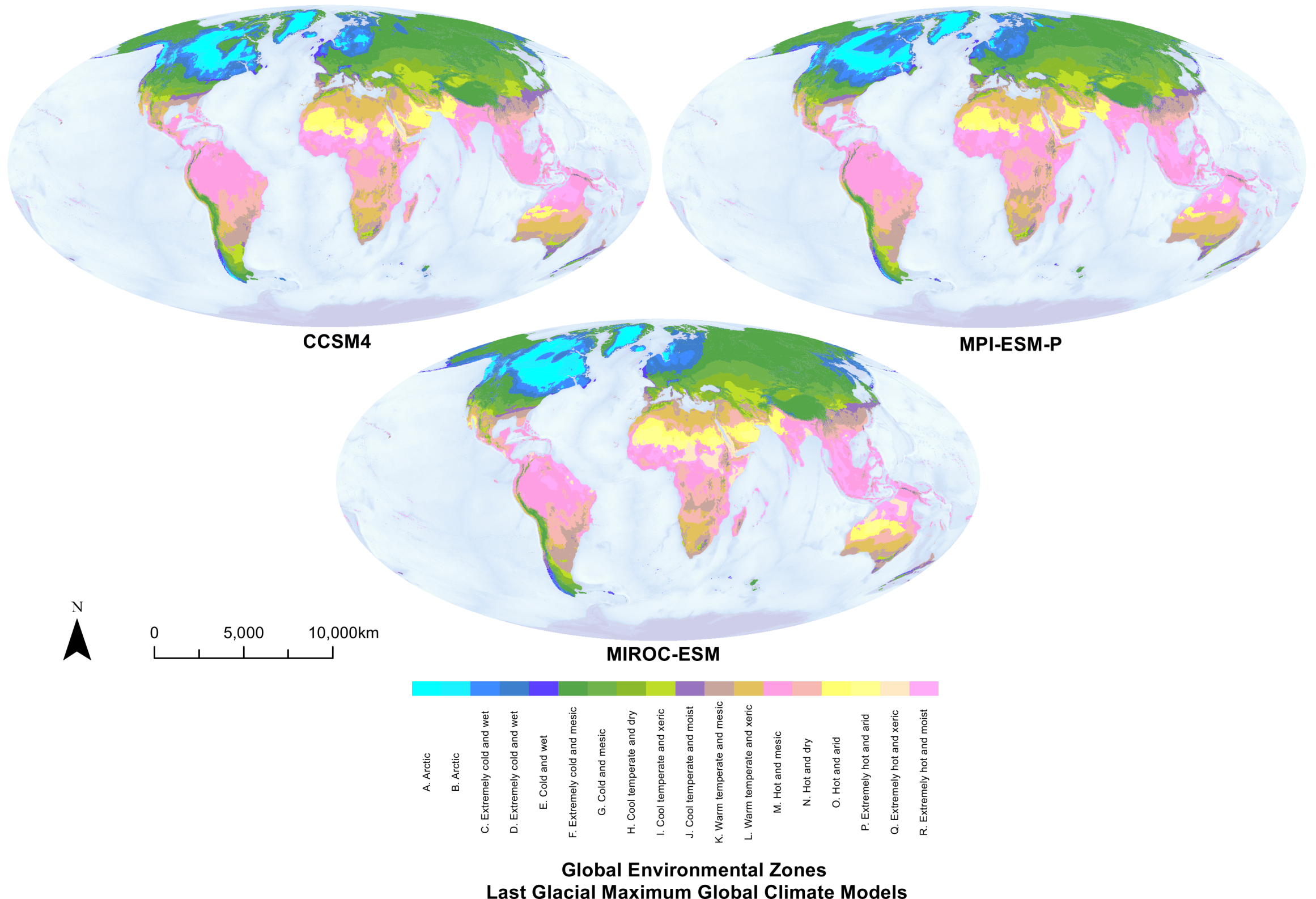


**Figure S1.3:** Global Environmental Zones for the Last Glacial Maximum, based upon three coupled general circulation models.

**Appendix S2 Reducing multiple global climate models to a single map output**

By using nine global climate models for the mid-Holocene, and three models for the Last Glacial Maximum, we capture some of the uncertainty portrayed in the climate data and MaxEnt modelling. It is advantageous however, to display a single modelled output of favourable climatic conditions for the lion during these time periods. We assign lion favourability categories to global environmental strata for each suitability model, and then apply the following rules to produce the final output.

- Lion favourability is assigned to the most commonly found category e.g. if two out of three models are ‘unsuitable’, we assign this category (modal winner).

Example:


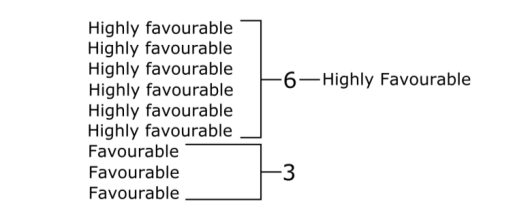


- If there is no modal winner, but there are two neighbouring classes with equal support over other classes, we split the favourability rating. So if four models are highly favourable, four models are favourable, and one model is suitable, then the category will be assigned as ‘favourable/highly favourable’.

Example:


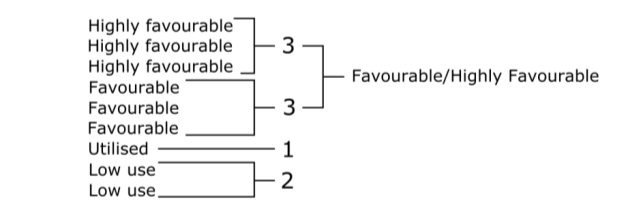


- If there is equal support for two classes that are not neighbouring, support for more than two classes, or no consensus between models, then the category is assigned to uncertain, given the wide discrepancy of results.

Example:


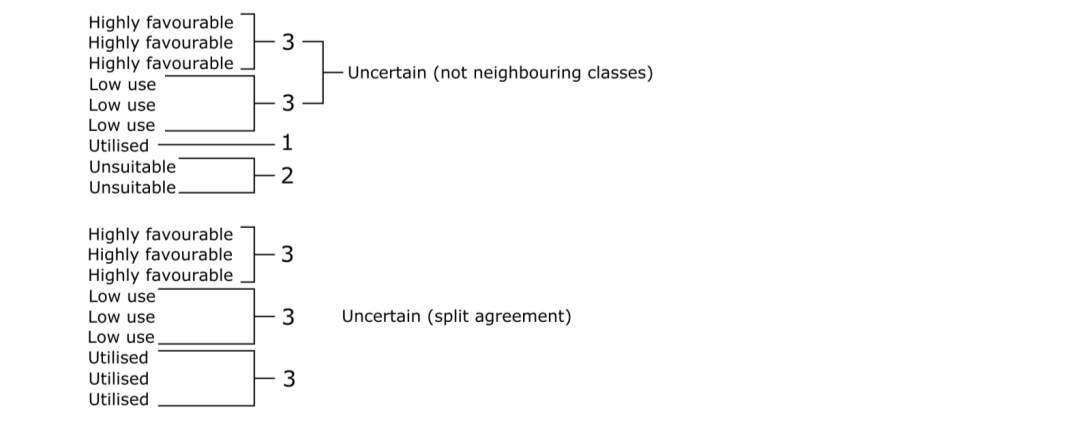


*References*

Soteriades, A. D., Murray-Rust, D., Trabucco, A. & Metzger, M. J. Understanding global climate change scenarios through bioclimate stratification. *Environ. Res. Lett.* **12,** 1–10 (2017).

**Appendix S3: MaxEnt Model Parameters and Results**

Final model parameters (using all localities)

- 1133 presence records used for training.
- 100000 points used to determine the Maxent distribution (background points).
- Environmental layers used (continuous):
  - ai_5m_ext
  - pet_sd_5m_ext
  - tm_dab05mext
  - tm_sd_5m_ext
  - Environmental layer extent: -19°E, 94°W, - 36°S, 50°N
- Regularization values: linear/quadratic/product: 0.050, categorical: 0.250, threshold: 1.000, hinge: 0.500
- Feature types used:
  - Hinge
  - Product
  - Linear
  - Threshold
  - Quadratic.
- Response curves: true
- Jackknife: true
- Remove duplicates: false
- Beta multiplier: 2.0
- Maximum background: 100000
- Add samples to background: false

Whilst the model displayed in Fig.1 is raw output, threshold values of bioclimatic suitability were necessarily obtained using logistic output.

Model validation

Models were run to ascertain Area under the Receiver Operator Curve (AUC) using 10 fold cross validation within MaxEnt under the same parameters as the final model. The average test AUC for the replicate runs was 0.923.

In addition, spatially independent cross validation was performed using the ENMeval package(Muscarella et al., 2014) within R(Team, 2015), using the ‘checkerboard2’ method. Parameters were consistent with those used in the final model. The model AUC score from spatially independent cross-validation was 0.818.

Raw Output and Model Thresholds


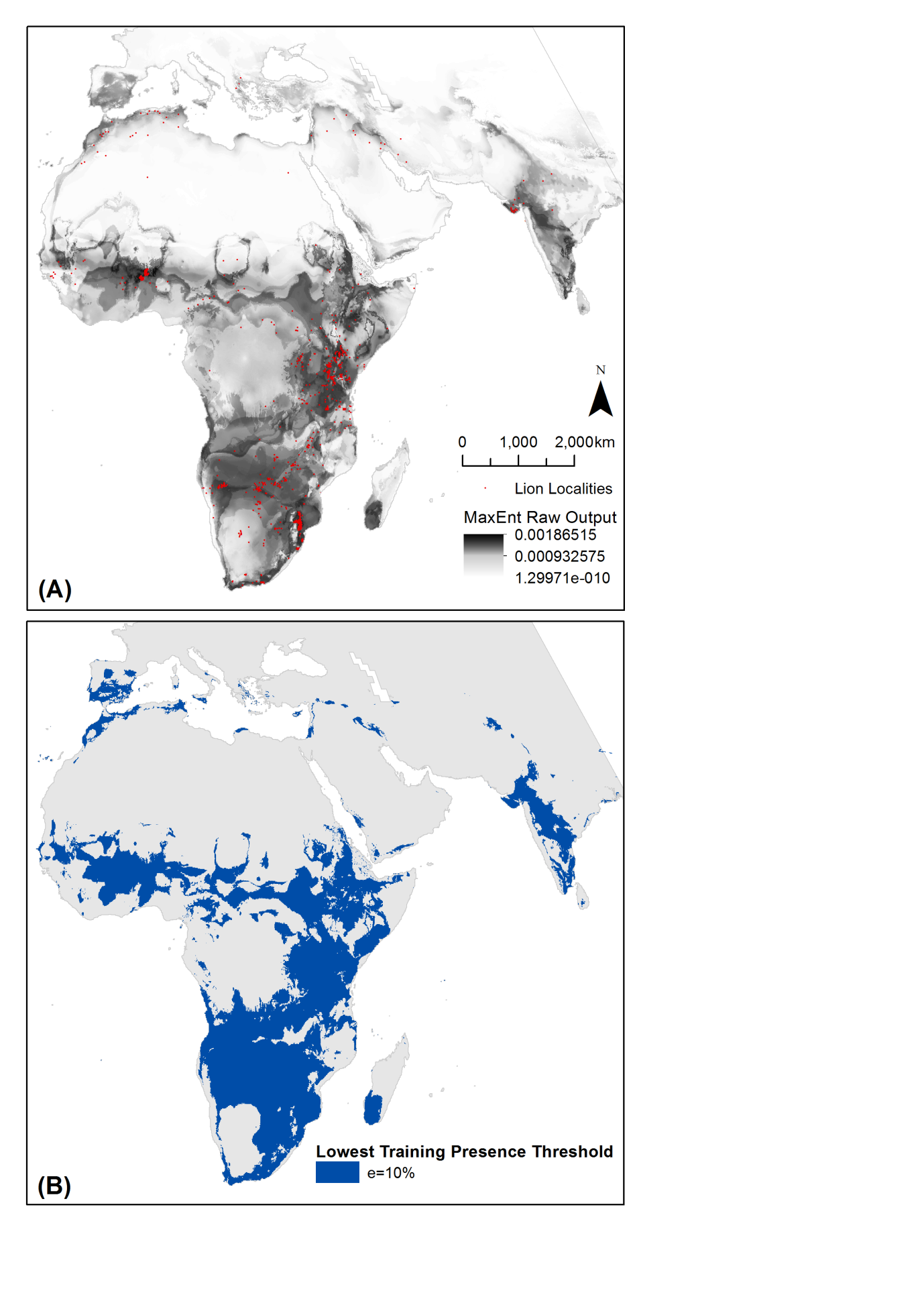


Figure S3.1: MaxEnt raw output (A) shows the present day suitability of Africa and the Near East for the lion based upon current and historic lion localities. We convert MaxEnt suitability into a threshold value (B) to enable comparison with Global Environmental Zones and Strata.

References

Muscarella R., Galante P.J., Soley-Guardia M., Boria R.A., Kass J.M., Uriarte M., & Anderson R.P. (2014) ENMeval: An R package for conducting spatially independent evaluations and estimating optimal model complexity for Maxent ecological niche models. *Methods in Ecology and Evolution*, **5**, 1198–1205.

Team R.C. (2015) R: A language and environment for statistical computing. .

**Appendix S4: All Lion Suitability Models for the Present Day, Mid-Holocene and Last Glacial Maximum**


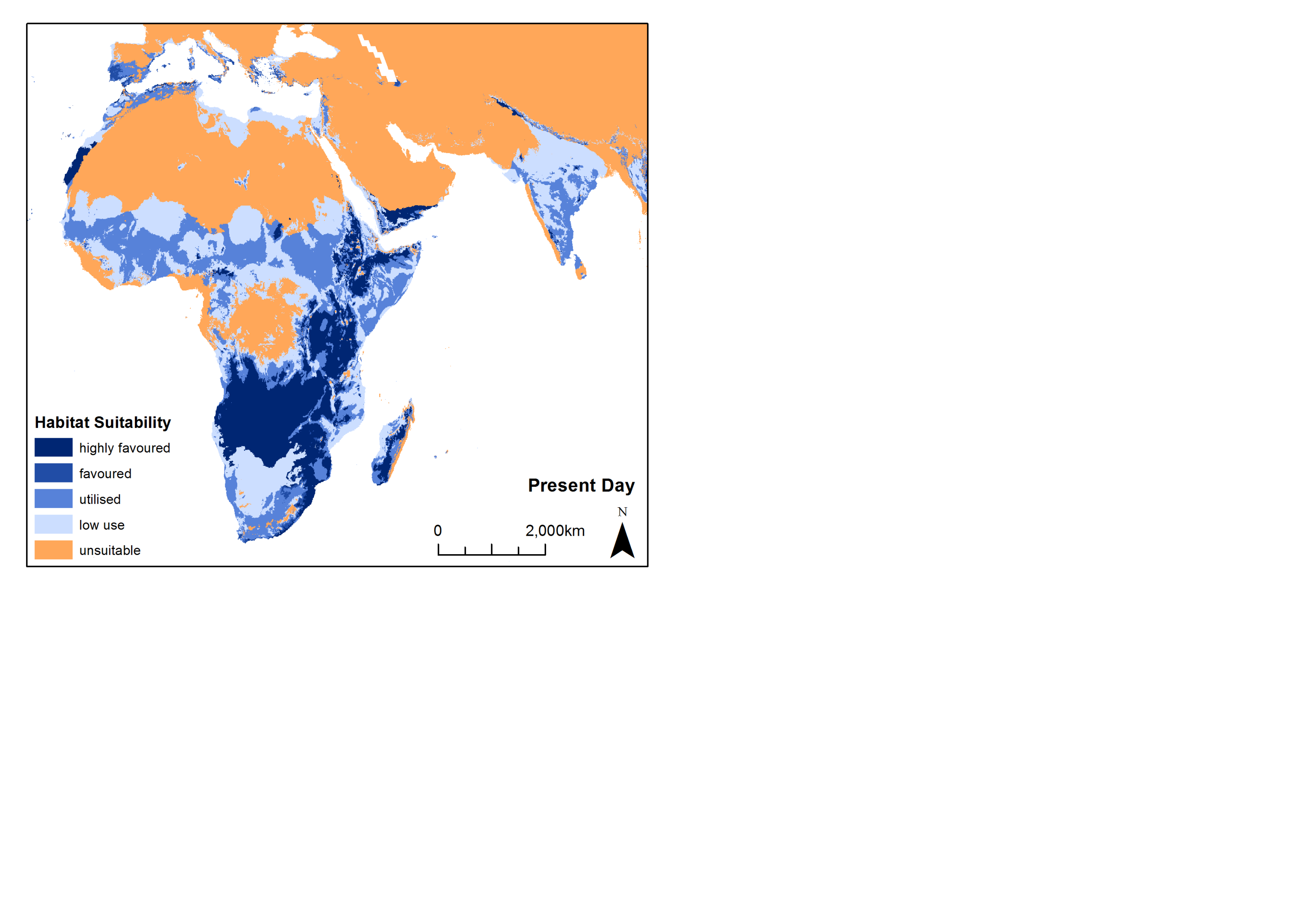


Figure S4.1: Present day lion suitability within the framework of Global Environmental Strata. Suitability is based upon both Equal Training Sensitivity and Specificity (ETSS) and Maximum Training Sensitivity plus Specificity (MTSS) MaxEnt species distribution model suitability thresholds.


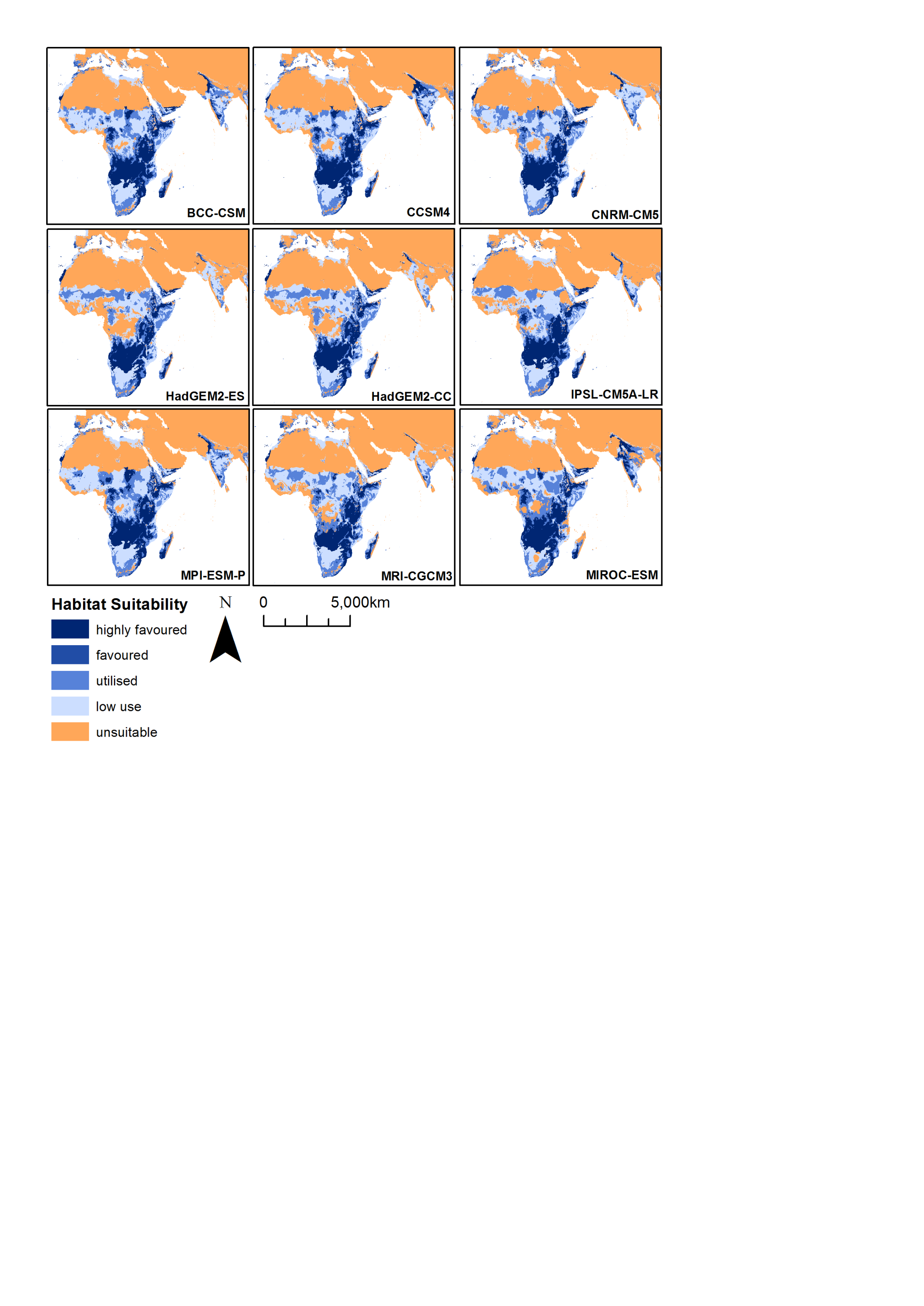


Figure S4.2: Mid-Holocene lion suitability presented for all climate model reconstructions of Global Environmental Strata (GEnS). Lion suitability is based upon both Equal Training Sensitivity and Specificity (ETSS) and Maximum Training Sensitivity plus Specificity (MTSS) MaxEnt species distribution model suitability thresholds.


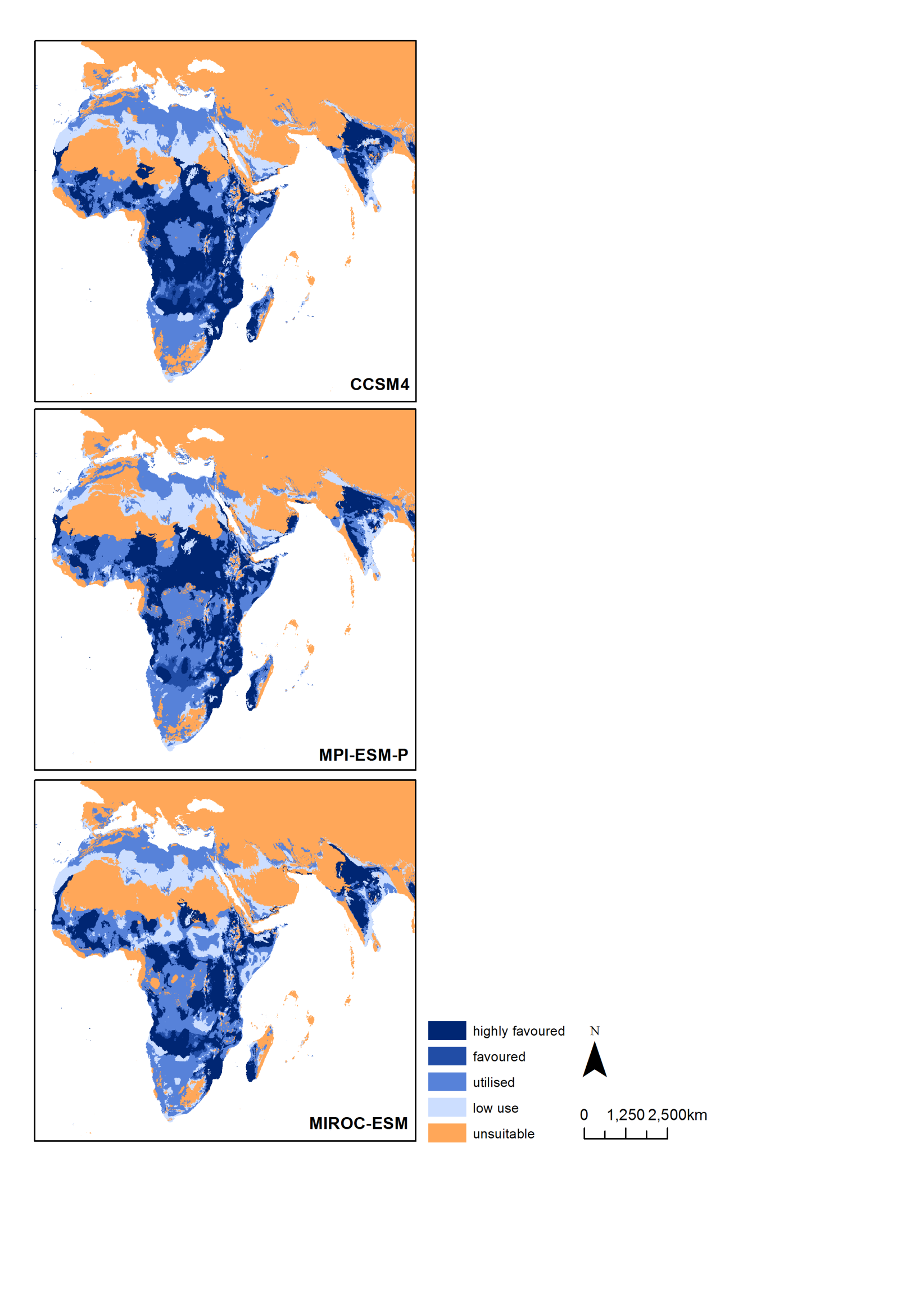


Figure S4.3: Last Glacial Maximum lion suitability presented for all global climate model reconstructions of Global Environmental Strata (GEnS). Lion suitability is based upon both Equal Training Sensitivity and Specificity (ETSS) and Maximum Training Sensitivity plus Specificity (MTSS) MaxEnt species distribution model suitability thresholds.

**Appendix S5 Saharan Rock Art reference table**

Table S5.1: Proxy evidence of lions within the Sahara during the African Humid Period ~12ka-6ka from depictions in African Rock Art. Other species present within the rock art, including wild species (bold).

| **British Museum**  **Reference** | **Country** | **Region** | **Rock Art Site** | **Other Species Present** |
| --- | --- | --- | --- | --- |
| 2013,2034.11784 | Libya | Tassili n Ajjer | Tadrart Acacus, Lion Rock |  |
| 2013,2034.4770 | Algeria | Tassili n Ajjer | Tadrart Acacus |  |
| 2013,2034.4788 | Algeria | Tassili n Ajjer | Tadrart Acacus |  |
| 2013,2034.4787 | Algeria | Tassili n Ajjer | Tadrart Acacus |  |
| 2013,2034.2000 | Libya | Tassili n Ajjer | Tadrart Acacus, Wadi Tihedene |  |
| 2013,2034.1726 | Libya | Tassili n Ajjer | Tadrart Acacus, Wadi Ineligghi | **Elephant** |
| 2013,2034.481 | Libya | Tassili n Ajjer | Tadrart Acacus, Acacus Mountains | **Hippopotamus** |
| 2013,2034.1469 | Libya | Tassili n Ajjer | Tadrart Acacus, Lion Rock | Sheep/goat |
| 2013,2034.11783 | Libya | Tassili n Ajjer | Tadrart Acacus, Lion Rock | Camel |
| 2013,2034.4759 | Algeria | Tassili n Ajjer | Tadrart Acacus |  |
| 2013,2034.11778 | Libya | Tassili n Ajjer | Tadrart Acacus, Lion Rock | Camel/**Giraffe** |
| 2013,2034.1464 | Libya | Tassili n Ajjer | Tadrart Acacus, Lion Rock | **Antelope** |
| 2013,2034.4763 | Algeria | Tassili n Ajjer | Tadrart Acacus | Cow/**buffalo**, **ostrich**, **roan antelope**, **giraffe** |
| 2013,2034.4864 | Algeria | Tassili n Ajjer | Oued Afar |  |
| 2013,2034.5006 | Algeria | Tassili n Ajjer | Oued Djerat |  |
| 2013,2034.12631 | Algeria | Tassili n Ajjer | Oued Djerat | Yes (undefined) |
| 2013,2034.5099 | Algeria | Tassili n Ajjer | Oued Djerat |  |
| 2013,2034.23850 | Algeria | Tassili n Ajjer | Tassili n'Ajer, Afara Plain | Unidentified animal |
| 2013,2034.2095 | Libya | Tassili n Ajjer | Tassili n'Ajer, Emi ‘n’ Eher | Cow |
| 2013,2034.4200 | Algeria | Tassili n Ajjer | Tassili n'Ajer, Tin Aboteka |  |
| 2013,2034.2084 | Libya | Tassili n Ajjer | Tassili n'Ajer, Emi ‘n’ Eher | Cow, horse, goat, horse, unidentified quadruped |
| 2013,2034.9166 | Niger | Aïr Mountains | Tanakom |  |
| 2013,2034.9165 | Niger | Aïr Mountains | Tanakom |  |
| 2013,2034.9158 | Niger | Aïr Mountains | Tanakom |  |
| 2013,2034.9164 | Niger | Aïr Mountains | Tanakom | **Giraffe**, **warthog**, unidentified quadruped |
| 2013,2034.9288 | Niger | Aïr Mountains | Tanakom | **Ostrich**, barbary sheep, unidentified animals |
| 2013,2034.9198 | Niger | Aïr Mountains | Tanakom | Cow, **ostrich**/**flamingo**, unidentified quadrupeds |
| 2013,2034.9257 | Niger | Aïr Mountains | Tanakom | Cow, **antelope**, **ostrich** |
| 2013,2034.9801 | Niger | Aïr Mountains | Iwellene |  |
| 2013,2034.9840 | Niger | Aïr Mountains | Iwellene |  |
| 2013,2034.9839 | Niger | Aïr Mountains | Iwellene | **Giraffe**, cow |
| 2013,2034.9862 | Niger | Aïr Mountains | Iwellene | **Ostrich** |
| 2013,2034.11033 | Niger | Aïr Mountains | Telahlaghe |  |
| 2013,2034.11287 | Niger | Aïr Mountains | Dabous |  |
| 2013,2034.10862 | Niger | Aïr Mountains | Dabous |  |
| 2013,2034.10861 | Niger | Aïr Mountains | Dabous |  |
| 2013,2034.10837 | Niger | Aïr Mountains | Dabous | **Antelope**, **giraffe** |
| 2013,2034.10859 | Niger | Aïr Mountains | Dabous | **Antelope**, **giraffe**, cow |
| 2013,2034.11128 | Niger | Aïr Mountains | Western Air |  |
| 2013,2034.10183 | Niger | Aïr Mountains | Mammanet | Giraffe |
| 2013,2034.9981 | Niger | Aïr Mountains | Mammanet | Unidentified quadruped |
| 2013,2034.10186 | Niger | Aïr Mountains | Mammanet | **Giraffe** |
| 2013,2034.9431 | Niger | Aïr Mountains | Tagueit | **Giraffe**, horse, **rhino** |
| 2013,2034.11125 | Niger | Aïr Mountains | Indakate | Horse, dog |
| 2013,2034.11115 | Niger | Aïr Mountains | Indakate | Horse, cow, **antelope** dog |
| 2013,2034.116 | Egypt | Wadi el-Obeid | Wadi el-Obeid |  |
| 2013,2034.111 | Egypt | Wadi el-Obeid | Wadi el-Obeid | Horse |
|  |  |  |  |  |

**Appendix S6: Modis Landcover Classes Table and GEnS/MODIS Comparison Figure**

Table S6.1: The relationship between each environmental strata and environmental zone is displayed, alongside the characteristic Modis natural land cover of each strata within our modelling extent. We include the percentage of each strata within each modelled lion habitat threshold, and within the IUCN extant distribution of the lion.

| **GEnS** | **GEnZ** | **Modis Natural Cover Characteristics** | **Lowest Presence Threshold, e=10%** | **IUCN Extant Distribution** |
| --- | --- | --- | --- | --- |
| 1 | A. Arctic | Neglible/No coverage | NA | NA |
| 2 | A. Arctic | Neglible/No coverage | NA | NA |
| 3 | B. Arctic | Neglible/No coverage | NA | NA |
| 4 | B. Arctic | Neglible/No coverage | 0.00 | 0.00 |
| 5 | B. Arctic | Neglible/No coverage | 0.00 | 0.00 |
| 6 | C. Extremely cold and wet | Neglible/No coverage | 0.00 | 0.00 |
| 7 | C. Extremely cold and wet | Neglible/No coverage | 0.00 | 0.00 |
| 8 | D. Extremely cold and wet | Neglible/No coverage | 0.00 | 0.63 |
| 9 | D. Extremely cold and wet | Neglible/No coverage | 0.00 | 0.06 |
| 10 | D. Extremely cold and wet | Neglible/No coverage | 0.00 | 0.00 |
| 11 | F. Extremely cold and mesic | Neglible/No coverage | 0.00 | 0.00 |
| 12 | F. Extremely cold and mesic | Neglible/No coverage | 0.00 | 0.00 |
| 13 | E. Cold and wet | Neglible/No coverage | 0.00 | 0.18 |
| 14 | E. Cold and wet | Neglible/No coverage | 0.00 | 0.29 |
| 15 | F. Extremely cold and mesic | Neglible/No coverage | NA | NA |
| 16 | F. Extremely cold and mesic | Barren_sparse and Grasslands | 0.00 | 0.00 |
| 17 | F. Extremely cold and mesic | Neglible/No coverage | 0.00 | 0.00 |
| 18 | F. Extremely cold and mesic | Neglible/No coverage | 0.00 | 0.00 |
| 19 | F. Extremely cold and mesic | Grasslands | 0.00 | 0.00 |
| 20 | F. Extremely cold and mesic | Grasslands and Barren_sparse | 0.00 | 0.00 |
| 21 | F. Extremely cold and mesic | Neglible/No coverage | NA | NA |
| 22 | F. Extremely cold and mesic | Neglible/No coverage | 0.00 | 0.00 |
| 23 | C. Extremely cold and wet | Neglible/No coverage | NA | NA |
| 24 | E. Cold and wet | Neglible/No coverage | 0.00 | 0.00 |
| 25 | F. Extremely cold and mesic | Neglible/No coverage | NA | NA |
| 26 | F. Extremely cold and mesic | Neglible/No coverage | 0.33 | 0.41 |
| 27 | F. Extremely cold and mesic | Grasslands and Barren_sparse | 0.00 | 0.00 |
| 28 | F. Extremely cold and mesic | Neglible/No coverage | NA | NA |
| 29 | F. Extremely cold and mesic | Grasslands | 0.00 | 0.00 |
| 30 | G. Cold and mesic | Grasslands | 0.00 | 0.00 |
| 31 | G. Cold and mesic | Neglible/No coverage | NA | NA |
| 32 | E. Cold and wet | Neglible/No coverage | 0.00 | 0.00 |
| 33 | G. Cold and mesic | Neglible/No coverage | NA | NA |
| 34 | G. Cold and mesic | Grasslands | 0.00 | 0.00 |
| 35 | G. Cold and mesic | Neglible/No coverage | 0.00 | 0.00 |
| 36 | G. Cold and mesic | Grasslands | 0.00 | 0.00 |
| 37 | G. Cold and mesic | Grasslands and Barren_sparse | 0.00 | 0.00 |
| 38 | G. Cold and mesic | Forest covers | 0.55 | 0.18 |
| 39 | E. Cold and wet | Neglible/No coverage | NA | NA |
| 40 | G. Cold and mesic | Grasslands and Forest Covers | 0.00 | 0.00 |
| 41 | G. Cold and mesic | Grasslands | 0.00 | 0.00 |
| 42 | G. Cold and mesic | Grasslands and Forest Covers | 2.72 | 0.85 |
| 43 | H. Cool temperate and dry | Grasslands | 0.00 | 0.00 |
| 44 | G. Cold and mesic | Forest covers and Grasslands | 0.00 | 0.00 |
| 45 | H. Cool temperate and dry | Grasslands and Barren_sparse | 0.71 | 0.00 |
| 46 | H. Cool temperate and dry | Neglible/No coverage | 0.00 | 0.00 |
| 47 | G. Cold and mesic | Forest Covers | 0.43 | 0.03 |
| 48 | G. Cold and mesic | Neglible/No coverage | 0.00 | 0.00 |
| 49 | J. Cool temperate and moist | Forest Covers | 4.86 | 0.86 |
| 50 | H. Cool temperate and dry | Grasslands, some Barren_sparse | 0.00 | 0.00 |
| 51 | H. Cool temperate and dry | Forest Covers and Grasslands | 0.00 | 0.00 |
| 52 | H. Cool temperate and dry | Neglible/No coverage | 0.00 | 0.00 |
| 53 | J. Cool temperate and moist | Neglible/No coverage | 0.00 | 0.00 |
| 54 | H. Cool temperate and dry | Grasslands, some Barren_sparse and Open Shrublands | 0.33 | 0.00 |
| 55 | J. Cool temperate and moist | Forest Covers | 0.51 | 0.12 |
| 56 | H. Cool temperate and dry | Neglible/No coverage | 43.98 | 0.00 |
| 57 | H. Cool temperate and dry | Grasslands and Forest Covers | 0.00 | 0.00 |
| 58 | I. Cool temperate and xeric | Grasslands, some Barren_sparse | 0.00 | 0.00 |
| 59 | I. Cool temperate and xeric | Grasslands, some Open_shrublands | 0.00 | 0.00 |
| 60 | J. Cool temperate and moist | Forest Covers | 2.55 | 0.44 |
| 61 | J. Cool temperate and moist | Forest Covers and Grasslands | 2.02 | 0.05 |
| 62 | J. Cool temperate and moist | Neglible/No coverage | 0.00 | 0.00 |
| 63 | I. Cool temperate and xeric | Grasslands and Barren_sparse | 0.00 | 0.00 |
| 64 | I. Cool temperate and xeric | Grasslands, some Open_shrublands | 0.00 | 0.00 |
| 65 | I. Cool temperate and xeric | Grasslands and Open_shrublands, some Barren_sparse | 9.49 | 0.00 |
| 66 | K. Warm temperate and mesic | Forest Covers, Grasslands and Woody_savannas | 6.02 | 0.29 |
| 67 | K. Warm temperate and mesic | Forest Covers | 10.26 | 0.92 |
| 68 | K. Warm temperate and mesic | Neglible/No coverage | 0.00 | 0.00 |
| 69 | I. Cool temperate and xeric | Barren_sparse, some Grasslands and Open_shrublands | 0.00 | 0.00 |
| 70 | K. Warm temperate and mesic | Open Shrublands and Grasslands, some Barren_sparse | 8.02 | 0.00 |
| 71 | K. Warm temperate and mesic | Woody_savannas, Grasslands, Forest Covers, Open_shrublands | 23.42 | 0.34 |
| 72 | K. Warm temperate and mesic | Neglible/No coverage | 0.00 | 0.00 |
| 73 | K. Warm temperate and mesic | Forest Covers | 12.16 | 1.64 |
| 74 | K. Warm temperate and mesic | Barren_sparse, Open_shrublands and Grasslands | 0.00 | 0.00 |
| 75 | K. Warm temperate and mesic | Open Shrublands, some Barren_sparse | 44.48 | 0.02 |
| 76 | K. Warm temperate and mesic | Woody_savannas and Grasslands | 50.46 | 1.42 |
| 77 | K. Warm temperate and mesic | Neglible/No coverage | 0.00 | 0.00 |
| 78 | L. Warm temperate and xeric | Open_shrublands, some Barren_sparse | 46.14 | 0.05 |
| 79 | L. Warm temperate and xeric | Barren_sparse and Open_shrublands | 0.21 | 0.00 |
| 80 | K. Warm temperate and mesic | Forest Covers | 6.41 | 0.14 |
| 81 | K. Warm temperate and mesic | Forest Covers, Savannas and Woody_savannas | 42.63 | 10.08 |
| 82 | L. Warm temperate and xeric | Woody_savannahs, Grasslands and Savannas | 66.04 | 1.53 |
| 83 | N. Hot and dry | Neglible/No coverage | 0.00 | 0.00 |
| 84 | L. Warm temperate and xeric | Open_shrublands, some Barren_sparse and Grasslands | 48.22 | 1.70 |
| 85 | N. Hot and dry | Forest Covers | 1.04 | 0.00 |
| 86 | L. Warm temperate and xeric | Barren_sparse and Open_shrublands | 5.39 | 0.00 |
| 87 | N. Hot and dry | Forest Covers, Savannas and Woody_savannas | 50.15 | 10.66 |
| 88 | N. Hot and dry | Savannas and Woody_savannas, some Grasslands | 88.37 | 9.20 |
| 89 | L. Warm temperate and xeric | Open_shrublands and Barren_sparse | 36.49 | 2.36 |
| 90 | N. Hot and dry | Neglible/No coverage | 13.82 | 0.00 |
| 91 | N. Hot and dry | Open_shrublands and Barren_sparse, some Savannas | 26.38 | 4.39 |
| 92 | N. Hot and dry | Barren_sparse, some Open_shrublands | 1.91 | 0.00 |
| 93 | N. Hot and dry | Forest Covers, some Woody_savannas | 7.98 | 1.61 |
| 94 | N. Hot and dry | Woody_savannas and Savannas, some Grasslands | 81.18 | 21.41 |
| 95 | M. Hot and mesic | Forest Covers | 8.16 | 0.00 |
| 96 | N. Hot and dry | Barren_sparse, Open_shrublands and Grasslands | 26.06 | 13.72 |
| 97 | N. Hot and dry | Woody_savannas and Savannas | 87.20 | 28.22 |
| 98 | M. Hot and mesic | Forest Covers and Woody_savannas | 46.60 | 1.37 |
| 99 | O. Hot and arid | Barren_sparse, some Open_shrublands | 0.76 | 0.00 |
| 100 | M. Hot and mesic | Savannas, some Barren_sparse, Open_shrublands, Grasslands and Woody_savannas | 82.84 | 37.44 |
| 101 | M. Hot and mesic | Woody_savannas, some Savannas and Forest Covers | 80.55 | 12.87 |
| 102 | M. Hot and mesic | Forest Covers, some Woody_savannas | 1.47 | 0.00 |
| 103 | O. Hot and arid | Barren_sparse, some Open_shrublands | 1.98 | 0.00 |
| 104 | M. Hot and mesic | Savannas, Woody_savannas, Barren_sparse, Grasslands and Open_shrublands | 80.56 | 32.37 |
| 105 | M. Hot and mesic | Woody_savannas and Forest Covers, some Savannas | 41.85 | 11.14 |
| 106 | M. Hot and mesic | Forest Covers, some Woody_savannas | 5.41 | 0.00 |
| 107 | O. Hot and arid | Barren_sparse, some Open_shrublands | 0.55 | 0.00 |
| 108 | R. Extremely hot and moist | Woody_savannas and Forest Covers, some Savannas | 12.72 | 18.28 |
| 109 | R. Extremely hot and moist | Savannas, Woody_savannas, Open_shrublands, Grasslands and Barren_sparse | 59.71 | 33.97 |
| 110 | P. Extremely hot and arid | Barren_sparse, some Open_shrublands | 0.79 | 0.00 |
| 111 | R. Extremely hot and moist | Forest Covers, some Woody_savannas | 3.92 | 0.00 |
| 112 | R. Extremely hot and moist | Forest Covers, some Woody_savannas | 3.41 | 6.99 |
| 113 | R. Extremely hot and moist | Woody_savannas and Savannas | 28.53 | 19.06 |
| 114 | R. Extremely hot and moist | Forest Covers, some Woody_savannas | 0.98 | 0.00 |
| 115 | R. Extremely hot and moist | Forest Covers and Woody_savannas | 1.38 | 0.00 |
| 116 | P. Extremely hot and arid | Barren_sparse, some Open_shrublands | 1.69 | 0.00 |
| 117 | R. Extremely hot and moist | Forest Covers, some Woody_savannas | 1.55 | 0.00 |
| 118 | R. Extremely hot and moist | Savannas and Woody_savannas, some Forest Covers | 26.45 | 15.10 |
| 119 | Q. Extremely hot and xeric | Grasslands, Open_shrublands and Savannas, some Woody_savannas | 38.47 | 15.94 |
| 120 | R. Extremely hot and moist | Forest Covers and Woody_savannas | 0.00 | 0.00 |
| 121 | Q. Extremely hot and xeric | Barren_sparse, some Open_shrublands and Grasslands | 0.12 | 0.02 |
| 122 | Q. Extremely hot and xeric | Savannas and Woody_savannas | 47.04 | 13.84 |
| 123 | Q. Extremely hot and xeric | Savannas, Grasslands and Open_shrublands, some Barren_sparse | 53.49 | 17.37 |
| 124 | Q. Extremely hot and xeric | Barren_sparse | 3.65 | 0.01 |
| 125 | Q. Extremely hot and xeric | Barren_sparse and Grasslands, some Open_shrublands | 24.21 | 1.68 |
|  |  |  |  |  |
|  | Highly Favoured |  | >80 |  |
|  | Favoured |  | >60 |  |
|  | Utilised |  | >40 |  |
|  | Low Use |  | >10 |  |
|  | Unsuitable |  | <10 |  |


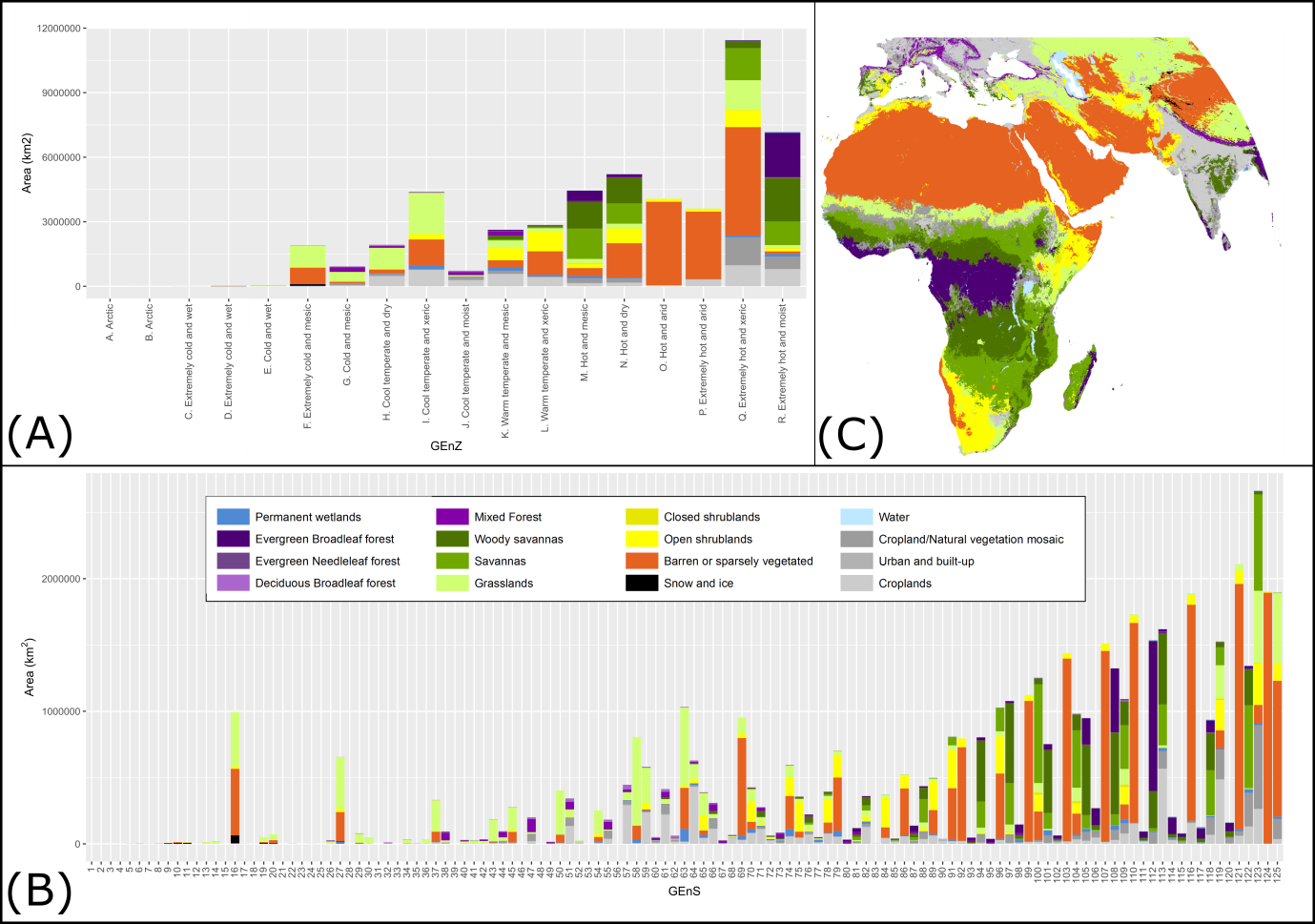


Figure S6.1: We display the proportion of both global environmental zones (A) and environmental strata (B) within the study extent (C) for the present day. Colours represent the MODIS landcover classes which characterise the zones and strata within the study extent. By comparing MODIS landcover to environmental zones and strata for the present day, we gain insight into the probable landcovers these bioclimatic categories represent for the mid-Holocene and Last Glacial Maximum. Croplands, Urban and built-up and Cropland/Natural vegetation mosaics are represented by greys, and are not useful in determining past land covers.
